# A Two-Branch ResNet-BiLSTM Deep Learning Framework for Extracting Multimodal Features Applied to PPG-Based Cuffless Blood Pressure Estimation

**DOI:** 10.3390/s25133975

**Published:** 2025-06-26

**Authors:** Zenan Liu, Minghong Qiao, Yezi Liu, Jing Zhang, Ling He

**Affiliations:** College of Biomedical Engineering, Sichuan University, Chengdu 610065, China; liuzenan@stu.scu.edu.cn (Z.L.); 2022223100048@stu.scu.edu.cn (M.Q.); 2022141670110@stu.scu.edu.cn (Y.L.); jing_zhang@scu.edu.cn (J.Z.)

**Keywords:** cuffless blood pressure estimating, photoplethysmography (PPG), physiological signals, deep learning, feature extraction

## Abstract

Cardiovascular disease is a major health threat closely associated with blood pressure levels. While continuous monitoring is essential, traditional cuff-based devices are inconvenient for long-term use. Current methods often fail to balance deep learning capabilities with interpretability, limiting further accuracy improvements. To address this problem, we propose a novel two-branch deep learning framework combining Residual Networks (ResNet) and Bidirectional Long Short-Term Memory (BiLSTM) for photoplethysmography (PPG)-based cuffless blood pressure estimation. The ResNet branch processes 60 features selected by Support Vector Machine-Recursive Feature Elimination (SVM-RFE) from manually extracted features, including our newly proposed trend features, while the BiLSTM branch processes complete PPG waveforms. Testing on 220 waveform segments from 218 patients in the MIMIC-IV dataset, our method achieves mean absolute errors of 3.47 mmHg and 2.81 mmHg, with standard deviations of 5.06 mmHg and 4.11 mmHg for systolic and diastolic blood pressure. This performance meets the Association for the Advancement of Medical Instrumentation (AAMI) standards and achieves an A rating according to British Hypertension Society (BHS) standards.

## 1. Introduction

Hypertension is a significant cardiovascular disease factor impacting billions of individuals globally [1], resulting in left ventricular hypertrophy, coronary and valvular heart disease, cardiac arrhythmias (including atrial fibrillation), stroke, and renal failure [2]. Consequently, the continuous monitoring of blood pressure is imperative for the early prevention and daily management of hypertension [3].

Conventional blood pressure measurement techniques rely on cuff-based methods, such as oscillometric and auscultatory approaches, which are unsuitable for continuous monitoring due to their intermittent operation and the discomfort they cause. Although invasive intravascular measurement is considered the clinical gold standard, its invasive nature confines its use primarily to intensive care settings. These limitations have driven research efforts toward non-invasive, cuffless methods for continuous blood pressure monitoring.

Photoplethysmography (PPG) has emerged as a promising non-invasive technique for blood pressure estimation. PPG sensors are already integrated into many wearable devices, making them ideal for continuous monitoring applications. Recent studies [4,5,6,7] have demonstrated that PPG-based blood pressure estimation can achieve clinically acceptable accuracy when combined with advanced signal processing and machine learning algorithms. Moreover, regulatory standards such as AAMI [8], BHS [9], and the emerging IEEE 1708 provide guidelines to ensure the reliability and safety of PPG-based devices. Research in PPG-based blood pressure estimation has followed two main approaches: (1) feature engineering with traditional machine learning and (2) end-to-end deep learning.

In the feature engineering approach, researchers extract predefined features from PPG signals, sometimes in combination with electrocardiogram (ECG) signals or pulse arrival time (PAT), and apply regression techniques such as multivariate linear regression, support vector machine (SVM), regression trees, random forests, and artificial neural networks [10,11,12,13,14]. While this approach offers interpretability, it often fails to capture the complex temporal dynamics of cardiovascular systems, resulting in limited accuracy that rarely meets the standards of the Association for the Advancement of Medical Instrumentation (AAMI) and British Hypertension Society (BHS). This is due to the fact that the PPG signal is a temporal signal, and temporal features capture time-dependent features in the PPG signal, such as changes in waveforms, periodic patterns, etc. These temporal characteristics are critical for understanding the dynamics of blood pressure, as they elucidate the immediate state and long-term trends in the cardiovascular system. Conversely, predefined static features are incapable of fully reflecting these changes, resulting in limited accuracy in blood pressure prediction.

With the development of deep learning, it has become possible to automatically extract detailed and comprehensive features from complete PPG waveforms by leveraging the hidden layers within the internal structure of deep learning models. In this way, feature selection is performed autonomously by the learning machine itself, without extracting predefined features [15]. Convolutional Neural Networks (CNNs) and Long Short-Term Memory (LSTM) networks have been used in many studies [16,17,18]. These studies have demonstrated notable improvements in accuracy compared to traditional methods. However, the “black box” nature of deep neural networks significantly limits their interpretability, which is essential in medical applications.

The research gap lies in the trade-off between accuracy and interpretability. Current methods often face a compromise between these two aspects. On one hand, manually engineered features are used, which prioritize interpretability. On the other hand, deep learning approaches are employed, known for achieving higher accuracy but often at the expense of interpretability. To address this limitation, we propose a novel hybrid approach that combines the strengths of both methodologies.

Our “Residual Networks–Bidirectional Long Short-Term Memory (ResNet-BiLSTM) two-branch framework” integrates manually extracted features with those automatically extracted from complete PPG waveforms. One branch takes a large set of manually extracted features as input, while the other processes the full PPG waveform. This integration mitigates the respective shortcomings of both approaches, resulting in improved accuracy while preserving interpretability.

The main contributions are highlighted as follows:

1. We propose seven novel groups of trend features designed to capture the signal trend over time. These features have not been previously utilized in the extraction of PPG signal features, but they are instrumental in PPG-based models that aim to predict blood pressure. Given the established correlation between the PPG waveform and the cardiac cycle, as well as blood pressure fluctuations, it is imperative to effectively capture the signal’s volatility and instantaneous frequency changes. These characteristics indirectly reflect the closely related relationship between the cardiac activity cycle and blood pressure fluctuations, thereby enhancing the time-dependent modeling capability of the model.
2. We implement a feature selection method using Support Vector Machine-Recursive Feature Elimination (SVM-RFE), which eliminates the features that are least correlated with blood pressure levels in each round of training, retaining only the features that are most critical for prediction.
3. We propose a two-branch deep learning framework combining ResNet and BiLSTM that simultaneously processes manually extracted features and raw PPG waveforms. This framework achieves both high accuracy and interpretability, with performance exceeding AAMI, IEEE 1708 standards and achieving BHS Level A standards, particularly for systolic blood pressure (SBP) prediction.

The structure of this paper is organized as follows. Section 2 presents related work in the PPG-based cuffless blood pressure estimation. Section 3 provides a detailed description of the various components of the proposed method. Section 4 details the experiments. Section 5 presents the results obtained from the proposed network, comparing the model with other BP estimation schemes in the literature. Section 6 concludes this work and discusses our future work based on the findings of this paper.

## 2. Related Work

PPG-based blood pressure estimation methods can be broadly categorized into two approaches: feature-based methods using manually extracted features and end-to-end deep learning methods using complete PPG waveforms [19]. The former is less accurate and struggles to meet international standards such as AAMI, whereas the latter is more accurate and typically meets AAMI standards but lacks interpretability. Each approach has its unique strengths and limitations that provide inspiration for our proposed hybrid framework.

### 2.1. Feature-Based Methods

Feature-based methods rely on extracting predefined physiological features from PPG signals and applying various regression techniques to estimate blood pressure. These methods offer interpretability but often struggle with accuracy and generalizability.

In early research, Kurylyak et al. [20] pioneered the application of artificial neural networks for PPG-based blood pressure estimation, extracting 21 predefined features from PPG signals. They trained their model using heartbeat samples from the MIMIC database, providing a comprehensive representation of PPG signals and blood pressure variations. Meanwhile, their approach demonstrated good performance in terms of standard deviation (SD), achieving 3.80 ± 3.46 mmHg for SBP and 2.21 ± 2.09 mmHg for diastolic blood pressure (DBP). Its main limitation was the exclusive focus on time-domain features, which ignored frequency-domain information and failed to address inter-individual differences, thereby limiting its generalizability across diverse populations. Subsequently, Liu et al. [12] proposed an enhanced blood pressure estimation method by combining the conventional 21 time-scale PPG features with 14 additional features derived from the second derivative of the PPG signal (SDPPG). Using support vector regression (SVR) trained on the MIMIC II dataset, their approach effectively reduced feature redundancy and improved estimation accuracy by approximately 40% compared to traditional neural network methods relying solely on time-domain features. Specifically, their SVR-based estimator achieved mean absolute errors (MAEs) and relative mean square deviations (RMSDs) of 8.54 ± 10.9 mmHg for SBP and 4.34 ± 5.8 mmHg for DBP. This combined feature set captures vascular aging and arterial stiffness information embedded in the SDPPG waveform, thereby enhancing the robustness and precision of cuffless blood pressure estimation. However, neither method fully leveraged the temporal dynamic characteristics of PPG signals, which are crucial for accurate blood pressure estimation.

In contrast to complex multi-feature models, several researchers have demonstrated the effectiveness of minimalist feature-based approaches for blood pressure estimation. Khalid et al. [21] employed a rigorous feature selection process using multicollinearity analysis, ultimately focusing on three key PPG-derived parameters: pulse area, pulse rising time, and width at 25%. Utilizing these features, the regression tree algorithm achieved high accuracy in SBP with a mean difference and standard deviation of −0.1 ± 6.5 mmHg, and DBP with −0.6 ± 5.2 mmHg, meeting the ISO standards for noninvasive blood pressure measurement devices. These findings underscore the importance of selecting high-quality, non-redundant features over a larger quantity of parameters for accurate blood pressure estimation. Similarly, Li et al. [22] adopted a streamlined approach with only seven carefully selected features, including PTT and HR. Their innovative implementation of Bi-LSTM architecture with residual connectivity demonstrates how advanced deep learning techniques can compensate for limited feature inputs, achieving SD for SBP and DBP of 14.505 mmHg and 6.442 mmHg, respectively. Such prediction results using only a very small number of features struggle to achieve high precision.

Feature selection represents a critical challenge in PPG-based blood pressure estimation. Chowdhury et al. [23], in 2020, employed multiple selection techniques on a dataset of 219 subjects, achieving large SD errors of SBP and DBP, 9.29 mmHg and 5.54 mmHg, respectively, barely meeting the BHS level B standard. Their approach primarily focused on quantity—extracting 107 features across time, frequency, and statistical domains. Nishan et al. [24], in 2024, extracted a total of 46 time, frequency, and time-frequency domain features from PPG and its derivative signals, and the selected features based on relief were combined with the SVR model to achieve very good blood pressure prediction results. However, the robustness of the results was not fully demonstrated as the model was based on high-quality screened PPG data and was not validated on a publicly available dataset. Therefore, in this study, we fully consider the difficulties in feature selection in previous studies and use the SVM-RFE approach to select PPG-based multimodal features for predicting blood pressure.

The primary limitation of feature-based methods is their sensitivity to individual cardiovascular variations and temporal drift in accuracy, as noted by Su et al. [25]. Additionally, manually defined features may not capture the full complexity of the relationship between PPG signals and blood pressure.

### 2.2. End-to-End Deep Learning Methods

To improve the accuracy of feature-based methods, researchers have developed end-to-end deep learning approaches that automatically extract features from raw PPG waveforms. These methods typically achieve higher accuracy but at the cost of interpretability.

In 2022, Yen et al. [26] employed the full waveform as input, automatically extracted features from the PPG signal using a convolutional neural network (CNN), and then analyzed these features to estimate physiological parameters using a LSTM network. Their model achieved impressive accuracy with mean absolute errors (MAEs) of 2.54 ± 3.88 mmHg for SBP and 1.59 ± 2.45 mmHg for DBP, demonstrating that deep learning can effectively capture the complex relationships between PPG waveforms and blood pressure. Wang et al. [27] 2022 proposed a novel methodology for converting PPG signals into images via visibility graphs (VGs). They then extracted feature vectors using a pre-trained deep CNN and solved the relationship between feature vectors and reference blood pressure using ridge regression. They achieved mean errors of 0.00 ± 8.46 mmHg for SBP and −0.04 ± 5.36 mmHg for DBP, demonstrating that image-based representations of PPG signals can effectively capture features relevant to blood pressure estimation. In 2022, Kim et al. [28] presented a deep learning model, DeepCNAP, which integrates a residual architecture and an attention-based jump connection. This integration enables the model to incorporate contextual information from PPG waveforms and learn temporal dependencies, resulting in MAE of 3.40 ± 4.36 mmHg for SBP and 1.75 ± 2.25 mmHg for DBP. The self-attention layer significantly enhanced the accuracy and robustness of continuous arterial blood pressure (ABP) estimation by facilitating the learning of relationships between different signal morphologies. While these deep learning approaches demonstrate impressive performance, they typically lack the interpretability of traditional feature-based methods that incorporate clinical physiological knowledge, presenting a trade-off between accuracy and explainability in blood pressure monitoring systems.

### 2.3. Hybrid Approaches

The integration of manually extracted features with deep learning techniques represents a promising avenue that potentially combines the interpretability of feature-based methods with the accuracy of deep learning approaches. However, this approach remains relatively underexplored, with only a few studies [17,29] attempting to extract different levels of PPG features through various deep learning architectures.

Our work addresses this research gap by proposing a two-branch framework that explicitly combines manually extracted features processed through ResNet with raw PPG waveforms processed through BiLSTM. Unlike previous hybrid approaches that primarily focus on multi-level feature extraction within a single network, our method maintains separate pathways for interpretable features and raw signal processing, enabling both high accuracy and interpretability. This dual-pathway design allows the model to leverage domain knowledge embedded in handcrafted features alongside the pattern recognition capabilities of deep learning applied to raw signals.

## 3. Materials and Methods

### 3.1. Problem Statement

PPG-based blood pressure prediction can be expressed as a nonlinear regression problem. The input PPG sequence segments are denoted by X, and the output blood pressure values are denoted by Y. In this context, Y is defined as SBP and DBP. The objective is to train a model so that its output corresponds to the two BP-labeled values (SBP and DBP) of the PPG over time. The combination of inputs and outputs of the regression problem is hereby defined as (1). Here, $X^{n}$ denotes a PPG record, $p_{x}$ and $f_{x}$ denote the number of sample segments and the corresponding feature dimensions in the PPG record, respectively, and M denotes the total number of PPG records. The fundamental components $Y^{n}$ are the SBP and DBP values corresponding to each sample segment, $p_{y}$, which denotes the number of output values (SBP and DBP) for each PPG record and M also denotes the number of sample segments.

(1) $$H=\left\{(X^{n},Y^{n})|X^{n}\in {\mathbb{R}}^{p_{x}\times f_{x}},Y^{n}\in {\mathbb{R}}^{p_{y}\times 2},n=1,2,\ldots ,M\right\}$$

### 3.2. Overview

**Figure 1:**
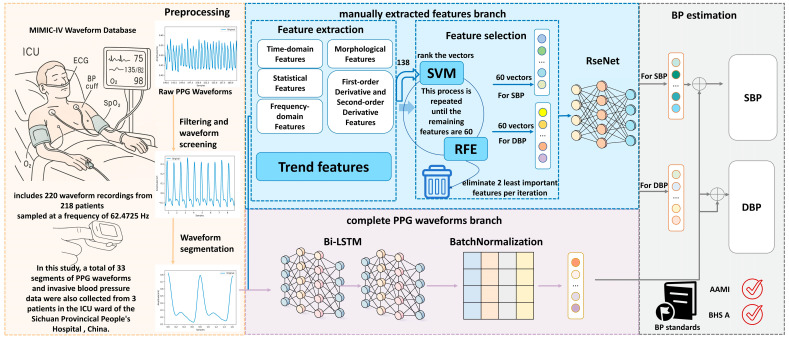
The general framework of the proposed method. It has four modules, (1) preprocessing, (2) manually extracted features branch, (3) completing the PPG waveforms branch, and (4) combining the outputs of two branches and output of the predicted blood pressure values.

In this study, we propose a ResNet-BiLSTM two-branch deep learning framework for PPG-based cuffless blood pressure estimation, as illustrated in Figure 1. The PPG waveforms are first pre-processed and then fed into two distinct branches. In the first branch, six groups of features totaling 138 manually extracted features are obtained from the processed waveforms. All these features are considered important as they comprehensively capture various physiological aspects of the PPG signal relevant to blood pressure estimation. Subsequently, the SVM-RFE method is applied for feature selection, reducing the number to 60 selected features, which are then input into the ResNet network. In the second branch, the pre-processed complete PPG waveforms are directly fed into the BiLSTM network. Finally, the outputs from both branches are fused through a concatenation layer, and the combined representation is passed through a fully connected layer to generate the final blood pressure prediction.

### 3.3. PPG Signal Preprocessing Based on Systolic Peak Detection

Ensuring the quality of the data is pivotal in the training of neural networks. Raw PPG signals are susceptible to unavoidable baseline drift and high-frequency noise, which typically originates from the standard acquisition process. In many previous studies [4,6,30], the first step in processing PPG signals is usually filtering to remove noise and baseline drift, ensuring signal quality. To address these issues, a fifth-order Butterworth bandpass filter with a passband of 0.5 Hz to 5 Hz is employed to effectively remove baseline drift and high-frequency noise. This frequency range is selected because the primary components of the PPG signal, which correspond to physiological phenomena such as heart rate and blood volume changes, are concentrated within this band. Frequencies below 0.5 Hz mainly represent baseline wander and motion artifacts, while frequencies above 5 Hz are typically dominated by high-frequency noise. Therefore, filtering within this range preserves the essential characteristics of the PPG waveform necessary for accurate analysis and blood pressure estimation. To mitigate the effects of discontinuities and saturated amplitudes, the PPG signal is partitioned into 10 s time segments. Specifically, for each 10 s segment of the PPG signal, if three consecutive sample points are found to have the same value, the segment is determined to contain discontinuous or saturated data and is removed.

Following this normalization, although waveform smoothing is achieved, incomplete cycles or missing waveforms may occur due to sensor contact problems. Therefore, further processing of the PPG data is necessary. Here, we refer to the anomalous segment removal method based on PPG systolic peak detection proposed by Elgendi et al. [31]. This process aims to remove signal segments containing anomalous data by detecting systolic peaks in the PPG signal to ensure the validity and accuracy of the data. Initially, the amplitudes of the PPG signal within all segments are truncated to remove values below zero. Subsequently, only signals with positive amplitudes are processed. Two convolution kernels are then defined for highlighting each cardiac cycle and PPG contraction peak-to-peak values, respectively. $f_{s}$ denotes the sampling frequency of the PPG, and $\omega_{1}$, $\omega_{2}$ represents artificially defined time variables that highlight each cardiac cycle and the peak of PPG contraction, respectively. In this study, these variables are set to 0.667 s and 0.111 s [32], respectively.

(2) $$C_{\mathrm{beat}}={\left[\frac{1}{\left(w_{1}*f_{s}\right)},\frac{1}{\left(w_{1}*f_{s}\right)},\ldots ,\frac{1}{\left(w_{1}*f_{s}\right)}\right]}_{w_{1}*f_{s}}$$

(3) $$C_{\mathrm{peak}}={\left[\frac{1}{\left(w_{2}*f_{s}\right)},\frac{1}{\left(w_{2}*f_{s}\right)},\ldots ,\frac{1}{\left(w_{2}*f_{s}\right)}\right]}_{w_{2}*f_{s}}$$

The application of these two convolution kernels to the filtered PPG segment results in the generation of two convolution signals, designated as $PPG_{beat}$ and $PPG_{peak}$, by which $PPG_{seg}$ represents the filtered PPG segment.

(4) $$PPG_{\mathrm{beat}}=PPG_{\mathrm{seg}}*C_{\mathrm{beat}}$$

(5) $$PPG_{\mathrm{peak}}=PPG_{\mathrm{seg}}*C_{\mathrm{peak}}$$

To further localize each contraction peak, threshold curves for the amplitude of the contraction peaks are calculated using the formula $TH_{\mathrm{peak}}=PPG_{\mathrm{beat}}+\alpha$, where $\alpha =\beta *z+PPG_{\mathrm{beat}}$,z is the arithmetic mean curve of $PPG_{seg}$, and in this study, the coefficient β is manually set to 0.03. The intervals containing the PPG contraction peaks are identified by comparing $PPG_{peak}$ and $TH_{peak}$, that is, $PPG_{peak}$ intervals that are larger than $TH_{peak}$.

The normal heart rate of an adult is typically between 60 and 100 beats per minute [33]. A 10 s PPG segment should contain a minimum of 10 cardiac cycles, defined as 10 peaks of contraction. However, it should be noted that the systolic peaks at the beginning and end of the segment may not always be fully captured. To address this potential issue, it is recommended to establish an upper limit of 8 systolic peaks. This approach ensures that only PPG segments with a minimum of 8 systolic peaks are retained, thereby helping to eliminate poor-quality segments from the PPG signal.

After that, a total of 13,321 10 s PPG fragments are obtained. In order to facilitate the subsequent feature extraction work, it is necessary to extract the PPG waveform of a single cycle from among the 10 s PPG segments. In this study, the windowing strategy proposed by Wang et al. [27] is adopted, with the aim of including a systolic peak before and after each cardiac cycle in order to capture the PPG signal and its trend for each cycle. Subsequent to this, the 10 s PPG segments are segmented, and a region of interest (ROI) is defined for every three PPG systolic peaks. Each PPG ROI encompasses a complete cardiac cycle, in addition to the downstroke of the preceding cycle and the upstroke of the subsequent cycle. The final dataset comprises 149,403 PPG segments, with the SBP and DBP values serving as labels. The distribution of the dataset complies with AAMI/ISO standards, as shown in Table 1.

### 3.4. Manually Extracted Features Branch

The PPG signal reflects the pulsatile blood flow within the vasculature extending from the heart to the extremities [34]. Consequently, the distinctive characteristics of PPG can provide valuable insights into arterial pressure. For example, PPG temporal features reflect the propagation of arterial pulse waves and vascular elasticity, which are closely associated with BP. Elevated BP typically results in shortened pulse transit time (PTT), decreased waveform rise time, and sharper systolic peaks, indicating increased arterial stiffness and altered hemodynamics [24,25,35,36,37]. In this study, we will extract six groups of 73 features, yielding a total of 138 feature vectors. Subsequently, the SVM-RFE method will be employed for the purpose of feature selection, aiming to identify the 60 feature vectors that exhibit the 60 most relevant feature vectors for blood pressure estimation. These feature vectors will subsequently be entered into the ResNet neural network.

#### 3.4.1. Feature Extraction

In several earlier studies, the pre-processed PPG signals were directly fed into the deep learning model, yielding favorable results. Nevertheless, utilizing solely the original waveform as the input results in a complex and computationally intensive neural network, which is not well-suited for everyday low-power wearable devices. Additionally, due to the “black-box” nature of the deep learning model, there is a lack of interpretability, which hinders further improvement of model performance. To address these challenges, numerous studies [24,25,35,36,37] have explored the extraction of diverse features from PPG signals, encompassing time-domain characteristics, morphological attributes, and statistical measures. In addition, some studies have investigated the extraction of frequency domain features, as well as first-order and second-order derivatives. Although the waveforms of single-cycle PPG cycles may vary from person to person, they are essentially the same in terms of characteristic features [38]. The incorporation of these features into the deep learning neural network enhances its scientific interpretability.

Building upon the extant literature, this study incorporates previously unexplored features. These features are then applied to the extraction of PPG waveform features, with the objective of effectively capturing the signal’s volatility and instantaneous changes. These features are particularly useful in capturing the correlation between the cardiac activity cycle and blood pressure fluctuation, providing insights into the dynamic behavior of the signal over time [39,40]. The following six sections provide a detailed exposition of these features: time-domain features, morphological features, statistical features, frequency-domain features, first-order derivative, and second-order derivative features, which have been defined in detail in numerous studies [24,25,35,36,37], and new extracted features named trend features.

##### Time-Domain Features

Time-domain features are those that are directly related to the physiological mechanisms of BP and are the most frequently used in the field of PPG signaling to predict BP. Cardiac output (CO) and peripheral vascular resistance (PVR) are the primary factors affecting blood pressure (BP = CO * PVR). The systolic width and diastolic width of the time domain features have been demonstrated to be directly related to peripheral vascular resistance in previous studies [41]. The time domain features selected for this study are enumerated in Table 2, items 1–31.

##### Morphological Features

Morphological features are determined through the precise segmentation and feature extraction of the waveforms, with a central focus on exploring the systolic and diastolic phases of the PPG waveforms. These phases correspond to the systolic and diastolic actions of the heart, respectively, along with the associated fluctuations in blood pressure. The morphological features selected in this study are delineated in items 32–37 of Table 2.

##### Statistical Features

**Figure 2:**
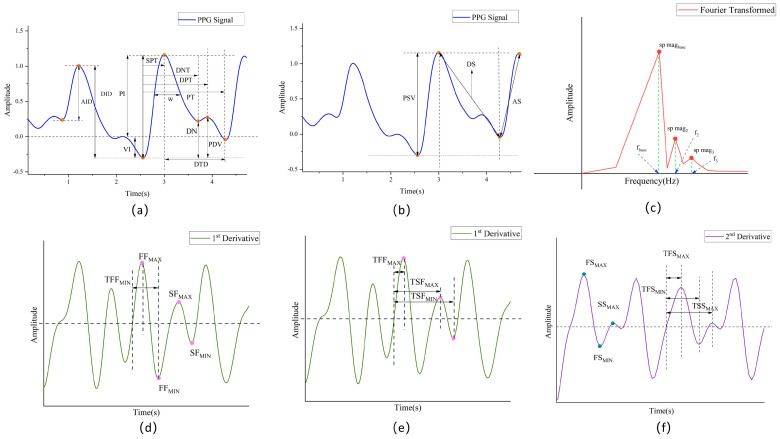
(**a**,**b**) Time-domain features and morphological features. (**c**) Frequency-domain features. (**d**,**e**) First-order derivative features. (**f**) Second-order derivative features.

As illustrated in Figure 2a,b, time-domain and morphological features reveal the local properties of the signal, thereby providing insight into immediate changes in cardiovascular activity. Conversely, global features reflect the trend and variability in the signal over a longer time span. These global features, inextricably linked to the long-term regulatory mechanisms of human blood pressure, offer a more comprehensive perspective on the subtle and macroscopic patterns in the PPG signals that may influence blood pressure changes. Consequently, they provide a richer set of information for the diagnosis and monitoring of cardiovascular diseases. The statistical features selected for the study are items 38–41 in Table 2.

##### Frequency-Domain Features

The PPG signal is transformed from the time domain to the frequency domain using a combination of the Fast Fourier Transform (FFT) and Short-Time Fourier Transform (STFT) to comprehensively analyze its spectral characteristics. For the FFT, frequency resolution is enhanced by zero-padding the signal to 16 times the next power of two, significantly improving spectral resolution even within 10 s windows. Additionally, STFT is applied to capture time-varying frequency components, enabling the extraction of instantaneous frequency and spectral entropy features. To minimize window truncation effects, complete pulse cycles are first identified through maxima and minima detection before performing frequency domain transformations. This approach effectively reveals the relationship between the PPG signal and the cardiovascular system in both static and dynamic frequency domains, as described in Equation (6). The frequency domain features selected for this study correspond to items 42–47 in Table 2, as illustrated in Figure 2c.

(6) HR=fundamental frequency×60 (beats per minutes,bpm)

##### First-Order Derivative and Second-Order Derivative Features

In some previous studies, it was found that some features of the first-order derivative and second-order derivative of the PPG signal can indicate the pulse wave index of cardiovascular disease risk, and its waveform features were similar to the ECG features in some parts [42,43,44]. The first-order derivative and second-order derivative features selected in this study are items 48–66 in Table 2, as shown in Figure 2d–f, respectively.

##### Trend Features

The seven groups of characteristics described below, which amount to 67–73 items in Table 2, effectively capture the volatility and instantaneous frequency changes in the PPG signal. These characteristics indirectly reflect the closely related relationship between the cardiac activity cycle and blood pressure fluctuations, thereby enhancing the time-dependent modeling capability of the model.

1. Short-Time Spectral Subband Energy Rate (STSSE)

The energy in the lower frequency bands may be associated with blood filling during ventricular diastole, whereas the energy in the higher frequency bands may be associated with rapid blood ejection during ventricular systole. To represent this correlation in a standardized numerical format that would allow the neural network model to recognize the mapping pattern between the energy characteristics of the PPG signal and blood pressure, we used STSSE.

The PPG signal is divided into five subbands: 0.5–1 Hz, 1–2 Hz, 2–3 Hz, 3–4 Hz, and 4–5 Hz. These subbands were chosen based on the typical frequency range of physiological components present in the PPG signal. The lower bound of 0.5 Hz corresponds to the minimum expected heart rate frequency, while the upper bound of 5 Hz captures the relevant higher-frequency components related to vascular dynamics. Dividing the signal into these subbands allows for a detailed analysis of the energy distribution across different frequency ranges, which is important for accurately characterizing blood flow and pressure variations. The proportion of the short-time energy of each subband in the total energy is calculated as an eigenvector, respectively. The STSSE is calculated as shown in Equations (7) and (8). $E_{i}$ is the energy of the ist subband. $|X(k){|}^{2}$ is the amplitude squared of the kth frequency point in the spectrum, representing the energy of that frequency point. ${\mathrm{STSSE}}_{i}$ is the energy rate of the ith subband, and N is the total number of subbands.

(7) $$E_{i}=\sum_{k=n_{i}}^{n_{i+1}-1}|X(k){|}^{2}$$

(8) $${\mathrm{STSSE}}_{i}=\frac{E_{i}}{\sum_{j=1}^{N}E_{j}},N=1,2,3,4,5$$

2. Root Mean Square Energy (RMSE)

Changes in blood pressure directly affect the strength of blood flow, which in turn affects the energy level of the PPG signal. Therefore, the energy level of the PPG signal is monitored using the RMSE. When blood pressure increases, the ejection force of the heart increases and the energy of the PPG signal rises accordingly [45], providing valuable information for blood pressure prediction.

The square root of the sum of the squares of all the samples in a frame is calculated to indicate the energy level of the frame. The formula for RMSE is shown in Equation (9).

(9) $$E_{\mathrm{RMS}}=\sqrt{\frac{1}{N}\sum_{n=0}^{N-1}x{\left(n\right)}^{2}}$$

3. Spectral Center of Mass

Since the frequency distribution of the PPG signal is associated with the systolic and diastolic phases of the heart, the trend in the concentration of the frequency distribution of the PPG signal correlates with the systolic phase of the heart as well as the diastolic phase as a ratio of the cardiac cycle [46]. A higher spectral center of mass implies an increase in the systolic ratio, and similarly, a lower spectral center of mass implies an increase in the diastolic ratio, providing potentially important clues for blood pressure prediction.

This is defined as the weighted average of the spectrum, with the weight being the magnitude of the spectrum. This calculation is indicative of the location of the “center of gravity” of the spectrum. The formula for the spectral center of gravity is shown in Equation (10), where C represents the spectral center of gravity, |X(k)| represents the spectral amplitude at the kth frequency, k is the index of the frequency, which depends on the sampling rate and the number of points of the FFT, as shown in Equation (11), and N is the length of the spectrum, i.e., the number of points of the FFT.

(10) $$C=\frac{\sum_{k=0}^{N-1}f_{k}|X(k)|}{\sum_{k=0}^{N-1}|X(k)|}$$

(11) $$f_{k}=\frac{k\cdot F_{s}}{N}$$

4. Spectral Bandwidth

The spectral centroid is indicative of the tendency of the PPG signal to concentrate in frequency; however, the degree of dispersion of the frequency distribution has not yet been thoroughly investigated. Blood pressure affects the homogeneity of blood flow, which in turn leads to changes in the degree of dispersion of the frequency distribution of the PPG signal. The spectral bandwidth reflects this dispersion, and the wider the spectral bandwidth, the greater the inhomogeneity of blood flow.

This represents the weighted average of the distances between all frequencies in the spectrum and the spectral center of mass. The formula for this calculation is presented in Equation (12), with all symbols defined in the preceding equation.

(12) $$B=\sqrt{\frac{\sum_{k=0}^{K-1}(f(k)-C{)}^{2}\cdot |X(k)|}{\sum_{k=0}^{K-1}|X(k)|}}$$

5. Overall Trend: In order to capture the overall trend change in the PPG signal, reflecting the change in vascular elasticity and thus the overall trend in blood pressure, the following three features were utilized: calculated amplitude, mean, and variance features.
6. Short-Time Energy (STE)

The heart functions as a pump, moving blood at a high velocity. This action is accompanied by the transmission of a substantial energy signal. The short-term energy of the PPG signal is a precise reflection of this rapid movement process.

The short-time energy is computed over a small time window to capture localized energy variations. The signal is segmented into multiple short-time frames, with each frame assigned a weight derived from a specific window function. In this study, the rectangular window is employed due to its ideal frequency response characteristics, minimal spectral leakage, and uniform weighting of all samples within the frame. This choice ensures that all data points in the segment contribute equally to the energy calculation, making it particularly effective for capturing abrupt changes in signal energy without introducing additional smoothing or distortion that other window types might cause. The calculation formula is shown in Equation (13), m where the frame index, which we set m to 20, N is the number of samples per frame set to 80 and $|x[n]{|}^{2}$ is used to calculate the instantaneous energy.

(13) $$E[m]=\sum_{n=mN}^{(m+1)N-1}|x[n]{|}^{2}$$

7. Short-Time Zero Crossing Rate (STZCR).

The over-zero points in the PPG signal are related to the systolic and diastolic cycles of the heart, so we use STZCR. STZCR can help to identify the transitions between these key points, indirectly reflecting the cardiac activity cycle, and providing additional time-dependent information for blood pressure prediction. This index is indicative of the frequency at which the signal intersects with the zero over a brief interval. The STZCR for the mth frame is calculated as shown in Equation (14), where the m-frame index, m, is set to 20, and N is the number of samples per frame, set to 80. The indicator function, denoted by 1{⋅}, returns 1 when the condition is valid and 0 when it is invalid. The variables x[n+mN] x[n+mN+1] denote the current sample and the next sample, respectively.

(14) $$ZCR[m]=\frac{1}{N-1}\sum_{n=0}^{N-2}1\{x[n+mN]\cdot x[n+mN+1]<0\}$$

#### 3.4.2. Feature Selection

In the previous feature extraction section, we extracted a total of 138 feature vectors, but these feature vectors may contain some irrelevant and redundant features. Therefore, it was crucial to perform feature selection. In this study, we employed a feature selection method known as SVM-RFE, which enabled us to systematically eliminate the least significant feature vectors. This process was conducted in a gradual and iterative manner, leading to a reduction in the number of feature vectors from 138 to 60. The reason for choosing 60 feature vectors rather than any other number is that we conducted extensive experiments, gradually reducing the number of feature vectors. Through the comparison of models 2–5 in Table 6, we can observe that retaining 60 feature vectors produces the best results. Based on our experimental results and experience, we determined that the optimal number of feature vectors for selection should be 60.

SVM-RFE is one of the most successful embedded methods in gene selection for cancer classification, proposed by Guyou et al. [47] Nowadays, it has been widely used in the field of gene selection and medical diagnosis. The SVM-RFE algorithm firstly trains the features by SVM according to Equation (15), where $y_{i}$ represents the SBP or DBP that we want to predict, $x_{i}$ is the individual feature vectors extracted in the previous step, and $a_{i}$ where is the Lagrange multiplier or support vector. Subsequently, the feature scores are calculated according to Equation (16), and the RFE algorithm searches for the lowest ranked features according to Equation (17) and subsequently eliminates these least important features in each iteration. In this study, we set the algorithm to eliminate two feature vectors per iteration and ultimately retained the next 60 feature vectors. The retained features for predicting SBP and DBP are shown in Table 2, respectively. In summary, the SVM-RFE algorithm first constructs a linear SVM model based on the input training dataset. Subsequently, the feature set is arranged in descending order according to its weights and the two features with the smallest weights are removed because they have less influence on the SVM. In the subsequent iteration, a new SVM is constructed based on the training dataset containing the remaining features. This process is repeated until the remaining features are 60. Regarding the distribution of these 60 selected features, approximately half are time-domain features, one quarter are derivative features, and the remaining quarter consists of trend features and frequency-domain features, both of which are derived from frequency domain analysis. Among the 60 feature vectors after feature selection, the seven newly proposed trend features are all applied, which also proves that the trend features we proposed are crucial for predicting blood pressure based on PPG signals.

(15) $$w=\sum_{i=1}^{n}a_{i}y_{i}x_{i}$$

(16) $$r_{i}={w_{i}}^{2}$$

(17) $$f=\arg\min(r_{i})$$

#### 3.4.3. ResNet Neural Networks

The feature vector left after feature selection will be input into the ResNet neural network after the fully connected layer and batch normalization process. ResNet was proposed by He et al. [48] in 2016, and the core idea is that each additional layer should more easily contain the original function as one of its elements. The application of ResNet to one-dimensional sequential signals leverages the advantages of residual connections, which effectively mitigate the vanishing gradient problem and enable the training of deeper and more expressive models. Although ResNet was originally designed for two-dimensional image data, recent studies have successfully adapted and applied ResNet architectures to various one-dimensional time-series signals, such as ECG [49,50] and sound [51,52], demonstrating strong performance and robustness.

**Figure 3:**
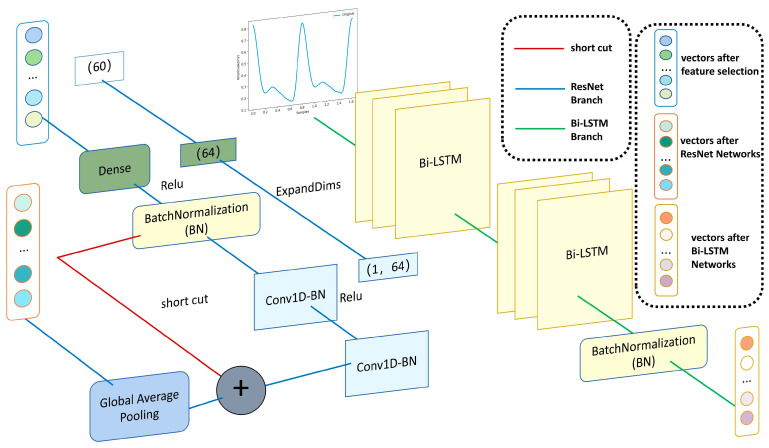
The detail of ResNet-BiLSTM networks.

The ResNet neural network used in this study primarily consists of two ResNet blocks, as illustrated in Figure 3. Each ResNet block includes a convolutional layer implemented with Conv1D, configured with 64 filters, a kernel size of 3, and a stride of 1. This is followed by batch normalization and a ReLU activation function before connecting to the next ResNet block. A shortcut connection directly adds the input tensor to the output of the convolutional layers. During training, this residual connection facilitates the direct flow of gradients from input to output, effectively mitigating the vanishing gradient problem commonly encountered in deep networks. Ideally, if the convolutional layers fail to learn useful features, they can simply pass the input through unchanged, effectively performing an identity mapping.

### 3.5. Complete the PPG Waveforms Branch

On top of the previous branch, we added the complete PPG waveforms branch. The sampling rate was 62.4725 Hz and the input sequence length was defined as 80, as the majority of the sequences in the dataset were less than 80 following preprocessing. Sequences that were shorter than 80 were padded with zeros, and sequences that were longer than 80 were truncated. This branch incorporated the full single-cycle PPG waveform into the Bi-LSTM, a configuration that has been demonstrated to be effective in previous studies [22,53,54,55]. Bi-LSTM has demonstrated strong effectiveness in predicting blood pressure from PPG signals. It overcomes the vanishing gradient problem inherent in traditional RNNs through its internal gating mechanisms, enabling the model to capture long-range temporal dependencies—an essential factor for stable blood pressure prediction. Our model employs two Bi-LSTM layers, each consisting of 100 cells. The first layer returns the full sequence output, while the second layer returns only the output at the last time step. A BatchNormalization layer is then applied to normalize the output of the second Bi-LSTM layer. The outputs from the two branches are concatenated along the feature dimension to form a combined tensor, which is subsequently passed through a fully connected (Dense) layer to generate the final prediction.

## 4. Experiment Setup

### 4.1. Dataset

MIMIC-IV Waveform Database: The MIMIC (Medical Information Mart for Intensive Care)-IV Waveform Database [56,57] is a database of a subset of the MIMIC-IV database [58] that contains critical care data from over 40,000 patients admitted to the intensive care unit (ICU) of Beth Israel Deaconess Medical Center (BIDMC). The waveform database includes 220 waveform recordings from 218 patients, including ECG, PPG, and invasive blood pressure signals, collected using bedside monitors in the intensive care unit. These signals were sampled at a frequency of 62.4725 Hz. While the MIMIC-II and MIMIC-III databases have been used in many previous studies of PPG prediction of blood pressure, MIMIC-IV improves upon MIMIC-III by including data updates and partial table reconstruction to accommodate evolving clinical research needs. To make the distribution of the dataset meet the AAMI/ISO specifications, we supplemented some data from MIMIC-III. The MIMIC database used in our study contains data collected from patients over multiple days and different time periods.

We also collected a total of 33 segments of PPG waveforms and invasive blood pressure data from 3 patients in the ICU ward of the Sichuan Provincial People’s Hospital. The PPG signal was captured by a fingertip Pulse Sensor with a sampling frequency of 96 Hz. This part of the data was used to further validate the validity of our model. The study was conducted in accordance with the Declaration of Helsinki, and the protocol was approved by the Medical Ethics Committee of Sichuan University (KS2022780) on [4 March 2022]. Informed consent for participation was obtained from all subjects involved in the study. Data management strategies were implemented to ensure the confidentiality, integrity, and security of the collected data, including anonymization of patient information, secure storage, and controlled access in compliance with institutional and ethical guidelines.

### 4.2. Training and Testing the Algorithms

We designed and validated deep learning models using the TensorFlow/Keras library in a Python 3.7 environment on a computer with an 8-core CPU (16 logical processors) and 16 GB of RAM. The graphics card used was an NVIDIA RTX 3060 to accelerate the model training process. Dividing the data into an 85% training set and a 15% test set, we applied the mean square error (MSE) as the loss function and set 0.001 as the initial learning rate of Adam’s optimizer. The model was trained for up to 150 epochs while using Early Stopping to prevent overfitting and preserve the optimal weights. The architecture combines a one-dimensional Residual Network (ResNet) block to process the feature vectors and a bi-directional LSTM layer to process the sequence data and finally outputs the predictions through a fully connected layer. The model contains 336 K parameters. Based on manual estimation, the total floating point operations (FLOPs) per inference was 51.35 million. The model’s inference latency was measured to be 58 ms per sample on an NVIDIA GeForce RTX 3060 GPU.

### 4.3. Evaluation Metrics

In the estimation of BP, AAMI [8] and BHS [59] criteria are widely used, with the AAMI requiring a mean error (ME) of no more than 5 mmHg and an SD of no more than 8 mmHg, and with the BHS criteria, the grading is determined by calculating the percentage of predicted samples for A, B, and C grades by calculating the percentage prediction of test samples with absolute errors less than 5 mmHg, 10 mmHg, and 15 mmHg, respectively. The consistency of the BP estimates with the true values was verified by Bland–Altman analysis. The metrics that we used are defined as follows:

(18) $$\Delta y_{i}=y_{i}-{\hat{y}}_{i}$$

(19) $$ME=\frac{1}{n}\sum_{i=1}^{n}\Delta y_{i}$$

(20) $$MAE=\frac{1}{n}\sum_{i=1}^{n}|\Delta y_{i}|$$

(21) $$\Delta {\bar{y}}_{i}=\frac{1}{n}\sum_{i=1}^{n}\Delta y_{i}$$

(22) $$SD=\sqrt{\frac{1}{n-1}\sum_{i=1}^{n}{\left(\Delta {\bar{y}}_{i}-\Delta y_{i}\right)}^{2}}$$

## 5. Results

In this chapter, we test our method using the previously mentioned evaluation metrics. Then, we compare our method with other methods and perform ablation experiments to further discuss our proposed method.

### 5.1. BP Estimation on Our Method Compared to the AAMI Standard and BHS Standard

Table 3 summarizes the results under the AAMI criteria. According to the AAMI standards, the mean error (ME) must not exceed 5 mmHg, and the SD must not exceed 8 mmHg. The BHS protocol assigns a grading of A–D to blood pressure devices based on the percentages of measurements that achieve an absolute difference of less than 5 mmHg, 10 mmHg, and 15 mmHg with respect to a gold-standard method, such as the intra-arterial monitoring used in the MIMIC-IV database. Only devices with a BHS of A or B can be recommended for clinical use. Table 3 illustrates the BP estimation on our method compared to BHS criteria.

As shown in Table 3, the ME of SBP and DBP in our results were −0.01 mmHg and 0.34 mmHg, respectively, which are much smaller than the 5 mmHg required by the AAMI standard. The SD were 5.06 mmHg and 4.11 mmHg, respectively, which are significantly smaller than the 8 mmHg required by the AAMI standard. As demonstrated in Table 4, the cumulative error in SBP measurements was less than 5 mmHg in 78.24% of cases, while for DBP, the corresponding figure was 85.00%, both of which exceed the A standards stipulated by the BHS. The proportion of SBP and DBP measurements exhibiting a cumulative error of less than 10 mmHg and 15 mmHg also far exceeds the British Health Standards Institution’s standard of Class A.

The IEEE 1708 standard for blood pressure measurement devices is largely derived from the well-established AAMI and BHS standards. We would like to emphasize that our results not only comply with the requirements set forth by AAMI and BHS but also meet the criteria specified in the IEEE 1708 standard. This alignment further validates the accuracy and reliability of our blood pressure prediction model, demonstrating its suitability for clinical and practical applications in accordance with recognized industry benchmarks.

Table 3 and Table 4 demonstrate that the blood pressure measurements derived from our method readily meet the two internationally recognized criteria, thereby substantiating the precision and practicality of our approach. The prediction of SBP exhibits superiority over DBP, a phenomenon observed in all existing PPG-based predictions of blood pressure. This is likely attributable to the notion that DBP is more stable than SBP. Concurrently, the MAE of both SBP and DBP in our results reached a maximum of 4 mmHg, thereby substantiating the efficacy of our algorithm in generating precise predictions for both SBP and DBP. As shown in Table 4, our algorithm easily met the requirements for class A devices in both SBP and DBP estimation.

In this study, we also collected a total of 33 segments of PPG waveforms and invasive blood pressure data from three patients in the ICU ward of the Sichuan Provincial People’s Hospital, China. The ME of SBP and DBP in our results were 0.977 mmHg and 3.15 mmHg, respectively, which are smaller than the 5 mmHg required by the AAMI standard; the SD were 6.24 mmHg and 7.02 mmHg, respectively, which are smaller than the 8 mmHg required by the AAMI standard.

### 5.2. Bland–Altman Analysis of BP Estimation on Our Method

**Figure 4:**
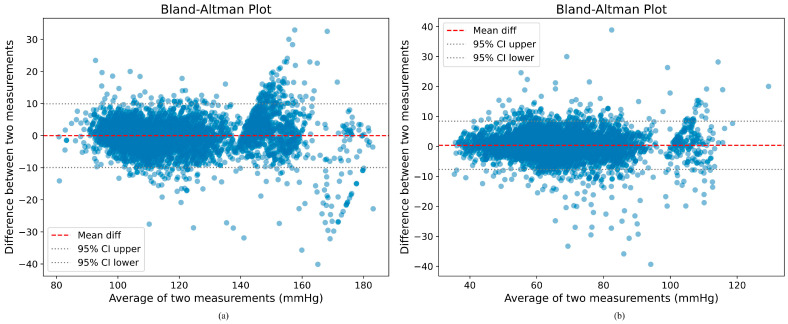
(**a**) Bland–Altman plot for SBP. (**b**) Bland–Altman plot for DBP.

As illustrated in Figure 4, the results of the Bland–Altman analysis on the test set are displayed.

The x-axis denotes the mean of the results for each sample, while the y-axis indicates the difference in measurements. The dashed lines, situated above and below the x-axis, represent the 95% confidence intervals, with ±1.96 times the SD. The red horizontal line denotes the mean difference. The majority of the data points fall within the acceptable range of ±1.96 SD, thereby further substantiating the reliability and stability of the method employed. A limited number of samples fall outside the confidence intervals, which may be attributable to the distinctive waveform characteristics and labeling values of these samples, with SBP and DBP values that were either excessively high or low. However, the number of samples falling outside the confidence intervals remained within acceptable limits, thereby substantiating the congruence between our predicted blood pressure values and the standard arterial blood pressure values measured intravascularly.

### 5.3. Regression Plot Analysis of BP Estimation on Our Method

**Figure 5:**
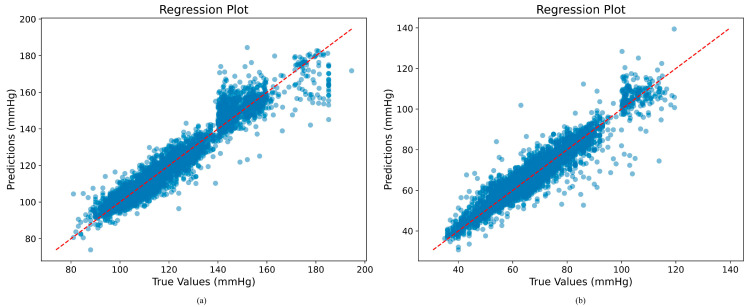
(**a**) Regression plot for SBP. (**b**) Regression plot for DBP.

As illustrated in Figure 5, the regression plots demonstrate a strong correlation between the estimated SBP and DBP values and the reference values.

These plots demonstrate a strong correlation between the majority of the estimated values and the actual BP values, with the line of best fit for the estimated values closely aligned with the ideal y = x (actual = estimated) line. The Pearson’s correlation coefficient between the SBP values predicted by our method and the reference measurements is 0.9633, while for DBP it is 0.9502. A limited number of data points deviated from the best-fit curve, particularly when SBP and DBP were either too low or too high. These aberrations could be attributed to the unique blood pressure status exhibited by certain patients under specific circumstances, such as the administration of antihypertensive medications within the ICU. However, the overwhelming majority of the samples exhibited a high degree of conformity to the best-fit curve, thereby underscoring the remarkable concordance between the blood pressure measurements obtained through our methodology and the gold standard blood pressure measurements.

### 5.4. Comparison with Other Works

In order to test the effectiveness of our method, we listed several methods applied on the MIMIC database and compared them, as shown in Table 5. The distinguishing characteristics among the various versions of the MIMIC database primarily pertain to the respective years in which the data were collected. MIMIC-IV represents the most recent database, with enhancements over MIMIC III, including updated data and modified table structures. To the best of our knowledge, there are currently no other studies in this field of research that use the MIMIC-IV database. However, this fact does not affect the results because all of the studies are based on data collected at the same hospital. The comparison includes the methods used for the databases used and the final results for each method. The evaluation metrics include SD and MAE.

As illustrated in Table 5, the MAE for SBP and DBP in our results were 3.47 mmHg and 2.81 mmHg, respectively, and the SD were 5.06 mmHg and 4.11 mmHg, respectively. Our method outperforms the results of the vast majority of studies. Only the latest study by Tian et al. slightly outperforms us on DBP, but we outperform them on SBP.

Kachuee et al. [63] employed a conventional machine learning approach and attained a satisfactory MAE of 6.34 mmHg and an SD of 8.45 mmHg for DBP. However, the outcomes for SBP were unsatisfactory, with an error value that was approximately twice that of DBP. This finding suggests that traditional machine learning methods, while effective in predicting DBP, perform poorly in predicting SBP.

Schlesinger et al. [46], N. Ibtehaz et al. [21], and C. Qin et al. [50] all employed CNN neural networks, and their results were significantly improved compared to traditional machine learning methods, confirming that deep learning methods are more suitable for the task of PPG prediction of blood pressure. While the accuracy of these approaches has been shown to significantly improve both SBP and DBP prediction, the prediction error of SBP remains substantial. Aguirre et al. [47] and Ibtehaz et al. [49] incorporated attentional mechanisms into their respective studies; however, the prediction of SBP remained unsatisfactory.

Stephanie Baker et al. [48] used a neural network model of CNN-LSTM and was able to achieve an MAE of 4.57 mmHg and an SD of 6.34 mmHg in the prediction of SBP, which is a significant improvement compared to the prediction of SBP in previous studies. This proves that the LSTM network model is very effective for improving the accuracy of predicting SBP in PPG-based blood pressure prediction, but their experimental results are still worse than the results of our method.

The latest study by Tian et al. [6] slightly outperformed us on DBP, but we outperformed them on SBP. This discrepancy may be attributable to the inherent characteristics of the utilized algorithm. Their algorithm demonstrated a propensity to prioritize precise DBP measurements, while ours placed a greater emphasis on achieving a balance between SBP and DBP measurements. From the evaluation metrics, all the models have better DBP prediction than SBP, but the gap between SBP and DBP is the smallest in our study, which indicates that our model is robust on both SBP and DBP and is more suitable to be used as an algorithm for measuring blood pressure. This further shows the superiority of our method.

The network architecture of Tian et al. [6] and Darbhasayanam et al. [4] is based on Transformers, which also demonstrates that the ResNet network we use is not inferior to other one-dimensional time series networks in processing one-dimensional time series signals.

### 5.5. Ablation Experiment

In this part, we verified the validity of each part of our proposed method. Firstly, we predicted blood pressure without feature selection and using only the manually extracted features branch; secondly, we predicted blood pressure after feature selection but using only the manually extracted features branch; and finally, we added the complete PPG waveforms branch for prediction and the results were shown in Table 6.

Although the results of Model 1 have not yet undergone feature selection, they exhibit satisfactory performance, indicating that all features are meaningful and do not lead to overfitting. The comparison between models 1 and 2 shows that performance in both SBP and DBP evaluation metrics improves after feature extraction. We employed the SVM-RFE feature selection method, which iteratively removes the two lowest-scoring features in each training round and re-evaluates feature importance in subsequent rounds. This approach mitigates the influence of prior training on later feature importance assessments, thereby preserving features critical for blood pressure prediction from PPG signals while effectively filtering out those with minimal impact.

The results of Model 2 already demonstrate relatively strong performance, validating the effectiveness of the manually extracted feature branch. Our feature set includes time-domain, morphological, statistical, frequency-domain, first-order derivative, and second-order derivative features, as well as newly introduced trend features. These features collectively provide a comprehensive characterization of PPG signals from multiple perspectives, intricately linked to blood pressure generation, the cardiac cycle, and vascular elasticity. This multifaceted approach offers a nuanced understanding of the relationship between PPG signals and blood pressure.

Comparing models 2 and 6, we observe a significant improvement in SBP and DBP evaluation metrics after incorporating the complete PPG waveform branch, confirming the feasibility and accuracy of our dual-branch strategy. The Bi-LSTM network excels at capturing temporal dependencies in both directions, aligning well with the characteristics of PPG temporal signals. Consequently, integrating the complete PPG waveform branch enhances the accuracy of blood pressure prediction.

Table 2 shows that many of the proposed trend features are retained in the feature vector after selection, indicating their strong correlation with blood pressure and their significant contribution to PPG-based blood pressure prediction. These newly proposed trend features relate closely to various phases of the cardiac cycle and the blood pressure formation process. They complement features not extracted in previous studies, providing a more comprehensive understanding of the link between PPG signals and blood pressure. Furthermore, these features improve the model’s generalization ability and robustness in blood pressure prediction.

## 6. Discussion and Conclusions

In this study, we proposed a ResNet-BiLSTM network with a two-branch structure to address the existing PPG-based problem of predicting blood pressure. The existing challenges include poor generalization and low accuracy across individuals using only manual feature extraction, and the lack of interpretability of features extracted directly from the complete waveform using only deep learning models, which hinders its clinical adoption. To address these challenges, we proposed a two-branch ResNet-BiLSTM structure that integrates these two approaches. The manually extracted features branch extracts’ preset features from PPG waveforms and subsequently inputs them into the ResNet neural network following feature extraction. Meanwhile, the complete PPG waveforms’ branch inputs the complete waveforms directly into the Bi-LSTM network and finally merges the two branches to output the prediction results.

The results show that we predicted blood pressure while easily meeting the AAMI criteria, IEEE 1708 standard, and the BHS grade A criteria, while on the assessment metrics, our results were better than the listed previous studies. And there was a great improvement in solving the problem of the poor prediction accuracy of SBP. Through consistency experiments, we verified the consistency of our model prediction results with the gold standard. Through ablation experiments, we confirm the validity of the double-branching results and feature extraction in our method. By comparing the features left before and after feature extraction, we demonstrate the importance of our proposed new features for blood pressure prediction.

For deployment on low-power wearable devices, further optimization such as model pruning, quantization, or architecture simplification may be considered in the future to reduce computational cost and latency. In the future, we will work to validate our algorithms with more non-clinically collected dynamic PPG data and to optimize the network to be more lightweight for easy integration into wearable devices. The development of a solution to the problem of relying on pressure sensors to predict blood pressure in commercially available wearable devices is also underway.

## Figures and Tables

**Table 1:** The distribution of our dataset and the requirements of AAMI/ISO standards.

Category	The Distribution of Our Dataset	AAMI/ISO Requirements
SBP ≥ 160 mmHg	5.02%	≥5%
SBP ≥ 140 mmHg	21.22%	≥20%
SBP ≤ 100 mmHg	11.11%	≥5%
DBP ≥ 100 mmHg	5.13%	≥5%
DBP ≥ 85 mmHg	22.36%	≥20%
DBP ≤ 60 mmHg	30.58%	≥5%

**Table 2:** All the features extracted in this study. (SBP) means features extracted for the prediction of SBP; (DBP) means features extracted for the prediction of DBP.

Feature	Definition	Feature	Definition
1. PSV(SBP)(DBP)	Peak systolic value	38. SK(SBP)(DBP)	Skewness in one cycle
2. PDV(SBP)	Peak diastolic value	39. KU(SBP)(DBP)	Kurtosis in a cycle
3. DN(SBP)(DBP)	Dicrotic notch	40. VAR(SBP)(DBP)	Variance over a cycle
4. t_PI_(SBP)(DBP)	Pulse period	41. MF(SBP)(DBP)	The ratio of maximum value to mean square value
5. PDV/PSV		42. f_base_	Fundamental frequency
6. (PSV-PDV)/PSV		43. sp mag _base_(SBP)	Spectral magnitude at the fundamental frequency
7. DN/PSV(SBP)(DBP)		44. f_2_	Frequency of the second-largest spectral component
8. (PDV-DN)/PSV		45. sp mag_2_(SBP)(DBP)	Amplitude of the second-largest spectral component
9. SPT(SBP)(DBP)	Systolic peak time	46. f_3_(SBP)(DBP)	Frequency of the third largest spectral component
10. DNT(SBP)(DBP)	Dicrotic notch time	47. sp mag_3_(SBP)(DBP)	Amplitude of the third largest spectral component
11. DPT	Diastolic peak time	48. TFF_MAX_(SBP)(DBP)	Time of the first maximum value of the first derivative
12. DPT-SPT(SBP)(DBP)		49. TFF_MIN_(DBP)	Time of the first minimum value of the first derivative
13. width	Bandwidth in hemisystole	50. TSF_MAX_(SBP)(DBP)	Time of the second largest value of the first derivative
14. A_2_/A_1_(SBP)(DBP)	Area ratio of the ascending and descending branches of a waveform	51. TSF_MIN_(SBP)(DBP)	The time to the second minimum of the first-order derivative
15. SPT/PSV		52. FS_MIN_/FS_MAX_	
16. PDV/(t_PI_-DPT)(SBP)(DBP)		53. SS_MAX_/FS_MAX_	
17. SPT/t_PI_(SBP)(DBP)		54. (FS_MIN_+SS_MAX_)/FS_MAX_	
18. DNT/t_pi_(DBP)		55. TFS_MAX_(SBP)(DBP)	
19. DPT/t_pi_		56. TFS_MIN_(SBP)(DBP)	
20. (DPT-SPT)/t_pi_		57. TFF_MAX_/t_PI_(SBP)(DBP)	
21. VI	Valley depth	58. TFF_MIN_/t_PI_(SBP)(DBP)	
22. PT(SBP)	Cycle duration	59. TSF_MAX_/t_PI_(DBP)	
23. PI	Peak height	60. TSF_MIN_/t_PI_(SBP)(DBP)	
24. DTD(SBP)(DBP)	Decline duration	61. TFS_MAX_/t_PI_(SBP)(DBP)	
25. AID	Wave height	62. TFS_MIN_/t_PI_(SBP)(DBP)	
26. DID	Waveform depth	63. (TFF_MAX_-TFS_MAX_)/t_PI_(DBP)	
27. AS(SBP)(DBP)	Rising slope	64. (TFF_MIN_-TFS_MIN_)/t_PI_(SBP)	
28. DS(DBP)	Downward slope	65. (TSF_MAX_-DNT)/t_PI_	
29. K(SBP)(DBP)	The ratio of the mean value to the difference between the minimum and maximum values in a cycle	66. (TSF_MIN_-DPT)/t_PI_(SBP)(DBP)	
30. AA(SBP)	Descending area	67. STSSE(DBP)	Short-time spectral subband energy rate
31. DA(SBP)(DBP)	Rising area	68. RMSE(SBP)	Root mean square energy
32. AID[X]	Threshold points divided into 10 equal parts according to AIDs	69. spectral centroid(SBP DBP)	Spectral center of mass
33. SW[X](SBP)(DBP)	Time from the wave start point to each AID[x] threshold point	70. spectral_bandwidth(SBP DBP)	Spectral bandwidth
34. DID[X]	Threshold points divided into 10 equal parts according to DID	71. Overall Trend(SBP)	Calculate amplitude, mean, and variance characteristics
35. DW[X](SBP)(DBP)	Time from wave end point to each DID[x] threshold point	72. STE(SBP)(DBP)	Short-time energy
36. DAS[X](SBP)(DBP)	Sum of each DW[x] and corresponding SW[x]	73. STZCR(SBP)(DBP)	Short-time zero crossing rate
37. DDS[X](SBP)(DBP)	The ratio of each DW[x] to the corresponding SW[x]		

**Table 3:** BP estimation on our method compared to AAMI criteria. ME: mean error. SD: standard deviation. MAE: mean absolute errors.

SBP (mmHg)		DBP (mmHg)	
ME ± SD	MAE	ME ± SD	MAE
−0.01 ± 5.06	3.47	0.34 ± 4.11	2.81

**Table 4:** BP estimation on our method compared to BHS criteria. BHS: British Hypertension Society.

Cumulative Error	≤5 mmHg	≤10 mmHg	≤15 mmHg	Grade
BHS	60%	85%	95%	A
	50%	75%	90%	B
	40%	65%	85%	C
SBP	78.24%	95.02%	98.19%	A
DBP	85.00%	97.34%	99.17%	A

**Table 5:** Comparing our study with other studies. The results of our study are highlighted in bold to distinguish them from other studies.

Study	Dataset	Subject Sizes	Methods	MAE(mmHg)		SD(mmHg)	
				SBP	DBP	SBP	DBP
Schlesinger et al. [60]	MIMIC-II	106,074 30 s windows from 304 different patients	CNN Siamese	5.95	3.41	6.69	3.97
Aguirre et al. [30]	MIMIC-III	1131 subjects	seq2seq-attention	12.08	5.56	15.67	7.32
N. Ibtehaz et al. [27]	MIMIC-II	11,294 segments from 348 records	CNN	6.17	3.66	8.46	5.36
Stephanie Baker et al. [61]	MIMIC-III	222,343 “good” records	CNN-LSTM	4.57	3.36	6.34	4.96
Ibtehaz et al. [62]	MIMIC-III	127,260 episodes from 12,000 records	GRU+ Attention	5.73	3.45	10.69	6.86
C. Qin et al. [63]	MIMIC-II	47,964 segments	CNN	5.98	3.24	7.79	4.94
Darbhasayanam et al. [4]	MIMIC-III	11,787 segments	Vision Transformer	17.59	8.09		
Tian et al. [6]	MIMIC-III	808 subjects	PCTN	4.44	2.36	5.98	3.22
**Our Work**	**MIMIC-IV**	**149,403 segments from 220 records of 218 patients**	**ResNet-BiLSTM**	**3.47**	**2.81**	**5.06**	**4.11**

**Table 6:** Ablation experiment. The final results are highlighted in bold.

Serial Number	Method	MAE(mmHg)		SD(mmHg)	
		SBP	DBP	SBP	DBP
1	Manually Extracted Features Branch	4.58	3.45	6.12	4.84
2	Manually Extracted Features Branch+ Features Selection (60 feature vectors)	4.26	3.32	5.94	4.77
3	Manually Extracted Features Branch+ Features Selection (50 feature vectors)	4.50	3.38	5.99	4.79
4	Manually Extracted Features Branch+ Features Selection (40 feature vectors)	4.55	3.47	6.09	4.82
5	Manually Extracted Features Branch+ Features Selection (80 feature vectors)	4.47	3.38	6.03	4.80
**6**	**Manually Extracted Features Branch+** **Features Selection (60 feature vectors)+** **Complete PPG Waveforms Branch**	**3.47**	**2.81**	**5.06**	**4.11**

## Data Availability

The datasets used in this study are publicly available from the he MIMIC (Medical Information Mart for Intensive Care)-IV Waveform Database [56,57] which is a database of a subset of the MIMIC-IV database [58]. The database can be requested in the website: https://mimic.mit.edu (accessed on 11 May 2025).
